# Changes in Alcohol Behaviour among Adolescents in North-West Russia between 1995 and 2004

**DOI:** 10.1155/2012/736249

**Published:** 2012-09-25

**Authors:** Anastasiya Verho, Tiina Laatikainen, Erkki Vartiainen, Pekka Puska

**Affiliations:** Department of Chronic Disease Prevention, National Institute for Health and Welfare, Mannerheimintie 166, 00271 Helsinki, Finland

## Abstract

*Background*. Among Russian adults, alcohol consumption with binge drinking was high and increased during past decades. Little is known regarding adolescents' drinking. The present study investigates changes in alcohol-related behaviour among Russian youth between 1995 and 2004. *Methods*. A cross-sectional survey was conducted among the 15-year-old youths from all schools in Pitkäranta, Republic of Karelia, Russia. In 1995, 385 students participated (response 95%), in 2004—395 (response 85%). *Results*. The proportion of abstainers decreased: boys from 26% to 13% (*P* = 0.002), girls from 23% to 12% (*P* = 0.007). The age of first alcohol consumption decreased among both genders. First alcohol drinking with friends increased among boys from 65% to 79% (*P* = 0.031), among girls from 49% to 70% (*P* = 0.001). Weekly drinking increased: boys from 13% to 28% (*P* < 0.001), girls from 6% to 15% (*P* = 0.001). The prevalence on inebriation increased among girls from 45% to 60% (*P* = 0.012), beer consumption from 8% to 21% (*P* = 0.006) by 2004. Gender differences were less prominent in 2004. *Conclusion*. Negative changes: early drinking initiation and more frequent alcohol consumption were observed among Russian youth by 2004. Regular monitoring, effective policy measures, and health education are necessary to prevent further increase in alcohol consumption and subsequent burden of alcohol-related diseases in Russia.

## 1. Introduction

Adolescence is recognized as a critical period in the development of lifestyles, including alcohol drinking behaviour. Patterns of alcohol use from experimentation to more established ones develop in adolescence and young adulthood [1], besides increased association with mortality, accidents and injuries [2], exposure to alcohol interferes with normal social, and psychological and physiological development during adolescence [3, 4]. The contribution of the environment plays a major role in the initiation of alcohol consumption. Its interaction with certain genes exposes adolescents to a risk of further alcohol related disorders [1, 5].

Russia belongs to the so-called “Vodka belt” countries [6] with one of the highest global levels of spirit consumption in the world [7] and a high prevalence of binge drinking [8]. Country has undergone significant political and economical changes since “Perestroika”—the program of Mikhail Gorbachev which included country's political, economic, and social restructuring. The period is known as a beginning of market economy in the Soviet Union. This process has affected health behaviour among adults. Alcohol consumption has increased among both men and women [9] vanishing results of the antialcohol campaign of 1985 [10]. Over the last couple of decades official sales of all types of alcoholic beverages have increased, with the exception of spirits while relative income has increased [11]. Excessive alcohol consumption significantly affects mortality rates in Russia [12]. In 2006, the registered prevalence of alcoholism together with alcoholic psychosis were 1,513 per 100,000. Prevalence of alcoholism and related psychosis per 100,000 among 15–17 year olds increased from 846 in 1995 to 1982 in 2006 [13]. 

Little is known about changes in alcohol-related behaviour among Russian adolescents in recent decades. The available data only represents the Russian capital Moscow and is thus problematic for the extrapolation to the entire country [14, 15]. This study aims to reflect changes in alcohol consumption behaviour between 1995 and 2004—a transition period impacted by foreign investments in the beverage sector with aggressive marketing, among Russian youth from the region of Pitkäranta in the Republic of Karelia, which is a relatively rural area in northwestern Russia. 

## 2. Methods

This study was implemented as a part of a survey on cardiovascular risk factors among Pitkäranta's adolescents in 1995 and 2004 [16]. The study sample comprised all 15-year-old pupils from all 10 comprehensive schools in the city and region of Pitkäranta. In 1995, 385 children participated in the survey with a response rate of 95% and in 2004, 395 children participated with a response rate of 85%. Data were gathered using a self-administered questionnaire, with principally precoded answers, distributed in schools. The questionnaire has been described in detail in earlier publication [16]. The section of the questionnaire regarding alcohol comprised questions on: (a) existing experience of alcohol consumption, (b) first experience of drunkenness, (c) alcohol consumption among close relatives and friends, and (d) available social and commercial sources of alcohol supply. 

First experience of alcohol consumption and first experience of getting drunk were assessed through questions “in which grade did you try alcohol for the first time?” To reveal the social context where alcohol was experienced for first time, adolescents were asked if they tried alcohol for the first time with parents, friends, by themselves, or with somebody else. Frequency of alcohol consumption was assessed by asking participants how often they had been drinking during 12 months previous to the survey. In that question, due to a small number of frequent drinkers, answers daily, a couple of times a week, and once in a week are combined in a common category—weekly; a couple of times a month and once a month were combined into one category—monthly; three times and once a year or less were combined into one category—a few times a year or less. 

Prevalence of being drunk was studied among those with experience of alcohol consumption by asking “how many times during the previous 12 month did you experienced the following conditions after drinking alcohol: lost consciousness, were very drunk, were drunk, were slightly drunk, and were not drunk”. Due to the few answers in some categories (e.g. loss of consciousness was reported by 4 boys in 1995 and 3 in 2004 and 1 case among girls in 1995) on degree of drunkenness, answers “were slightly drunk”, “were very drunk”, and “lost consciousness” were combined into the category “felt drunk”. 

The preferred beverages were studied by a last week's recall on different beverages types consumed. Beverage types asked were beer, wine, spirits, and alcopops. Alcopops, that are flavoured, spirit-based, ready-made beverages, were included only in 2004 survey, since these were not prevalent in the Russian alcohol market in 1995.

The study was approved by the Ministry of Health and Social Development of the Republic of Karelia. Participation consent was obtained from participants and their parents. 

### 2.1. Statistics

Chi-square for dichotomous variables was applied to test differences in the prevalence, as well as gender differences, in the experience of alcohol consumption, period of consumption initiation, the frequency of drinking, and the social group where alcohol was experienced for the first time. The proportion of adolescents that had consumed each type of beverage during the week prior to the survey was calculated to reveal difference in preferences of drinks. All analyses were implemented separately for both genders using the statistical package SPSS, version 18.

## 3. Results

### 3.1. Existing Experience of Alcohol Consumption

The amount of participants who had never experienced alcohol drinking decreased, both among boys from 26% in 1995 to 13% in 2004 (*P* = 0.002) and among girls from 23% in 1995 to 12% in 2004 (*P* = 0.007). There were no statistically significant gender differences in either survey year on existence of experience of alcohol consumption (1995: *P* = 0.6; 2004: *P* = 0.9). 

### 3.2. First Experience of Alcohol Consumption

In 2004, among boys the first experience of alcohol consumption had taken place significantly at an earlier age compared to 1995. In 1995, only 23% of boys reported that they had consumed alcohol in 6th grade or earlier. The respective proportion in 2004 had increased to 36%. Among girls, the familiarization with alcohol was different. As with boys, the percentage using alcohol first in the 9th grade decreased from 1995 to 2004, but the percentage using first in the 6th grade also decreased; so that in 2004 the majority of girls (69%) had their first experience of alcohol in the 7th or 8th grade. In 2004, gender differences in the grade of first experience of alcohol consumption were statistically significant (*P* < 0.001) (Table 1).

### 3.3. Social Context of First Alcohol Experience

Friends appeared to be the main group with whom alcohol is first tried for both boys and girls. Among boys, 65% in 1995 compared to 79% in 2004 reported that they first tasted alcohol with friends. Respective proportions for girls were 49% and 70%. Parental involvement in experiencing alcohol for the first time among children decreased between 1995 and 2004, among boys from 22% to 15%, and among girls from 40% in 1995 to 25% in 2004. The importance of other social groups, such as relatives and neighbours, decreased as well. In both genders, changes were statistically significant. The difference between the genders regarding the social group for alcohol experience was statistically significant in 1995 (*P* = 0.008). Experiencing alcohol for the first time with parents was statistically significantly more prevalent among girls. (Table 2) Self-purchasing of alcohol increased between years of survey. In 1995, 36% of boys who had at least tried alcohol reported buying alcohol versus 48% in 2005 (*P* = 0.052). Among girls, these numbers were 16% in 1995 and 42% in 2004 (*P* < 0.001).

### 3.4. Frequency of Alcohol Consumption during the 12 Months prior to the Survey

Boys reported drinking more often than girls in Pitkäranta. Furthermore, alcohol consumption became more prevalent (*P* < 0.001) among boys in 2004 compared to 1995. The number of adolescents who did not consume alcohol at all almost halved from 33% to 17% between the survey years. Weekly drinking among adolescents more than doubled from 13% to 28%. Among girls, the frequency of alcohol consumption increased statistically significantly between 1995 and 2004 (*P* = 0.001), and weekly alcohol consumption increased from 6% to 15%. The amount of abstainers among adolescents decreased from 27% to 17% between the survey years (Table 3).

### 3.5. Feeling Drunk after Alcohol Consumption during the Past 12 Months prior to the Survey

The prevalence of youth who reported having been drunk during the past 12 months increased statistically significantly only among girls, from 45% in 1995 to 60% in 2004 (*P* = 0.012). Among boys the prevalence of being drunk after alcohol consumption did not exhibit a significant change, being 57% in 1995 and 67% in 2004. 

### 3.6. Type of Beverage Consumed during the Week prior to Survey

Beer was the most consumed drink by both genders in 2004. For boys this was followed by vodka, alcopops, and wine. For girls it was followed by long drinks, wine, and vodka. The prevalence of consumption of alcoholic beverages during the survey years was higher among boys and did not explicit major changes except for the consumption of new beverages on the market such as canned long drinks. Among girls, beer consumption increased statistically significantly from 8% to 21% (*P* = 0.006) between 1995 and 2004 (Table 4).

## 4. Discussion

This study represents the changes in alcohol-related behaviour between 1995 and 2004 among adolescents in northwest Russia from a semirural area. Over the period surveyed, alcohol consumption increased among both boys and girls. The proportion of abstainers decreased among both genders. Among boys, the age of initiation of alcohol consumption decreased and in 2004, over one-third had their first experience in 6th grade (12 years old) or earlier. Among girls, the age of initiation increased slightly. Introduction to alcohol in the company with friends increased among both genders. The frequency of drinking increased among both genders with doubling in weekly consumption. Beer consumption dominates among both genders. Among boys, it is higher and did not change much since 1995. Among girls, it increased remarkably by 2004. The number of girls experiencing drunkenness after consumption of alcohol increased after 2004. 

A corresponding increase in the prevalence of alcohol consumption among both genders between 1993 and 2002 has also been reported in the Baltic countries. Alcohol intoxication in age of 13 or younger increased there as well [17]. The similar picture emerges from the WHO Health Behaviour among School Children Survey study conducted in Eastern European countries (Bulgaria, Croatia, Slovenia, The Slovak Republic, and Ukraine). There, the occurrence of first alcohol and intoxication experiences at age of 13 or earlier has increased as well. In the same study, Russia was represented only by St. Petersburg—the second largest city in Russia, where the occurrence of first alcohol and intoxication experiences increased as well [18]. In another study—the European School Survey Project on Alcohol and Other Drugs, where older age group from Moscow was presented, corresponding findings were obtained [14]. Conversely, in Japan a decrease in drinking prevalence among adolescents has been observed between 1996 and 2004 [19]. Also, in Finland, the amount of abstainers has increased among 12–18 year olds since 1997 [20].

Shared past commonalities in socioeconomic environment may have caused similarities between the alcohol-related behaviour of Russian and Easter-European youth, especially former Soviet-Baltic countries and Ukraine. In these countries, there has also been an increase in availability and selection of alcoholic beverages and they are also relatively cheap. Contrary, in Finland and Japan, strengthening of alcohol policies started to be implemented a long time ago and they are constantly monitored for adjustments [19, 20]. From 1973 to 1987, Finland experienced similar increase in adolescent drunkenness due to increase in overall alcohol consumption and alcohol availability together with liberalisation of alcohol policy in general [21].

Over the study period, the frequency of drinking among boys in Pitkäranta is almost double that of girls. However, between 1995 and 2004, the weekly alcohol consumption doubled among both genders. Also frequency of drinking among girls is still lower than that among boys, long-term consequences of existing increase should be considered. This might anticipate diminishing of gender differences in alcohol consumption is among Russian youth following a pattern observed in Canada and other countries participating in HBSC survey, by which there is near parity in the prevalence of alcohol consumption almost equal between the genders [15]. Since women are more vulnerable to health hazards caused by excessive drinking, future alcohol problems among women are likely to increase in the future, as it has been observed in Finland [22]. 

Parental alcohol consumption was not explored in our study, but in Pitkäranta. In Pitkäranta a tolerant attitude towards drinking in a child's close environment, such as by family and friends, may play a major role in increasing interest towards alcohol among youth in Pitkäränta, where the mean alcohol consumption has significantly increased among adults, especially among men between 1992 and 2002 [9]. It would be interesting to study whether in Russia the youth alcohol consumption culture corresponds to that of adults or whether it exists as a protest against societal norms. In the absence of clear studies to date, the extent to which early drinking initiation among Russian youth influences later drinking habits together with variations in alcohol drinking frequency and amount remains uncertain. 

Cultural and group components play an important role in determining drinking patterns [23, 24]. Among Pitkäranta's adolescents, peers play increased role. The introduction to alcohol increased among Pitkäranta's adolescents. This may reflect on changes in the macroenvironment, such as the greater availability of alcohol, access to it among youth [25], increased purchasing power, and weaknesses in implementing of antialcohol policy [26, 27].

 In Russia, the sale of alcohol to minors (under the age of 18 years) is banned, but there is no culture of comprehensively checking the buyers' age. Moreover the alcohol in Russia is both relatively cheap compared to other European countries and is widely available twenty-four hours a day in most places. Also purchasing power has increased since 1990 [9].

In our study, beer consumption showed remarkable increase among girls. Beer is reported to be the main alcoholic beverage consumed among adolescents worldwide [15]. Alcopops and other similar beverages have occupied their own market niche among both genders of Pitkäranta's adolescents. To date the alcohol concentration in such drinks in Russia can be upto 9%. Increasing popularity of alcopops among young people has been reported from other countries, especially among female adolescents [19, 28]. 

The prevalence of drunkenness among girls has increased almost to the boys' levels. However, it would be too early to assume that a modern drinking culture is taking over the traditional one in Russia since prospective studies are not available. It is possible that gender differences in drinking rates may diminish in Russia in the future. 

Overall, the observed changes in alcohol behaviour among young people in Pitkäranta correlate with those reported in Moscow also in older age groups and those among youth from other posttransition European countries [16]. Taking into account the effects of globalization and existing anti-alcohol legislation, it is, to some extent, possible to predict that future alcohol consumption trends among Russian youth will follow the direction of other Eastern European countries. 

The main possible factors behind negative changes in alcohol behaviour among Russian youth are weakly enforced, age limits on alcohol sales, low alcohol prices compared to other European countries together with affordable prices in relative to income [9], twenty-four hour alcohol availability, the inefficiency of preventive programs, the low level of alcohol harm-related literacy, and the existing familial drinking culture. Also, there are newer factors, such as new low-alcohol products that are introduced with aggressive marketing strategies, which raise alcohol consumption among adolescents. Recent increase in price affects only spirits thus further increase in consumption of other alcohol beverages could be expected. Due to observed changes in alcohol-related behaviour among youth and the growing alcohol sales, an increase in number of accidents, prevalence of sexually transmitted diseases, and unplanned pregnancies among adolescents could be expected. In the long term, the further increase in the burden of alcohol-related disease among adults could be expected in Russia.

## 5. Limitations and Strengths

Adolescents' self-reported drinking in cross-sectional surveys appears to be rather reliable, especially when closed-end questions such was the case in our study. A closed set of questions may aid in memorising occasions when alcohol was consumed, especially when different beverages were consumed [29]. Adults are likely to underreport especially the amount of alcohol drunk. In this study, we did not try to capture the amount of drinking, but rather other aspects as first experience and social contexts. Adolescence is also in rather alcohol experimenting phase and most likely such behaviour is not perceived embarrassing by youth [30]. Amongst others study's strengths were the sample representativeness (all adolescents of aged 15 years old from the same geographical area), the confidential participation and the high response rates in both survey years. However, its limitations should not be overlooked. The study comes also from one area of semi-rural Russia, but likely represents the trend among this kind of areas in Russia. Since the data represents only two observation points, it is difficult to conclude on more detailed dynamics of adolescents' alcohol-related behaviour between the survey years. The exact age of drinking initiation was not revealed, meaning that we were unable to assess the proportion of very early experiences in alcohol consumption. The exact amount of alcohol consumed prior to the survey week was not evident from our data. It would also have been interesting to study the self-attributed consequences of alcohol drinking as well as the exposure to alcohol marketing via traditional sources and the internet. 

## 6. Conclusions

Early initiation and more frequent alcohol consumption were observed among youth between 1995 and 2004. Changes in alcohol consumption among girls project further reduction in gender differences in alcohol consumption among Russian youth and, consequently, adults. Together with decreased number of abstainers and increase in alcohol consumption frequency, it urges for alcohol policy adjustments, based on regular monitoring of alcohol behaviour among Russian youth.

The regular monitoring of drinking patterns among youth is important for health policy making and public health perspectives. The systematic surveillance of alcohol behaviour among adolescent makes it possible to understanding trends in the prevalence of drinking among youth, detects weaknesses in anti-alcohol policy design and implementation, and helps initiate early preventive programs. The findings of the study suggest that health promotion programs should be updated and embedded in school curricula. The programmes should aim above all to postpone the initiation of alcohol consumption. 

It is most likely that in the future, drinking among Russians may cease to be an issue of a national identity but rather one of international homogenisation of the alcohol drinking culture, as in other European countries. Internationally recognized evidence-based alcohol control policies and actions should be implemented in the whole country alongside with comprehensive education on alcohol-related harm, taking into consideration the cultural nuances of Russia, targeting simultaneously both the youth and the adult population. 

## Figures and Tables

**Table 1:** First experience of alcohol consumption (only for alcohol consumers).

First experience of alcohol consumption	Boys				Girls			
	1995		2004		1995		2004	
	*N*	%	*N*	%	*N*	%	*N*	%
6th grade or earlier	30	23	53	36	29	20	18	12
7th grade	23	18	33	22	33	23	41	28
8th grade	37	28	41	27	39	27	60	41
9th grade	40	31	22	15	43	30	29	19

Total	130	100	149	100	144	100	148	100

Chi-square	*χ* ^2^ = 12.4 *P* = 0.006				*χ* ^2^ = 10.6 *P* = 0.014			

Gender differences in first experience of alcohol consumption: 1995 (*χ* ^2^ = 1.25, *P*-value = *ns*); 2004 (*χ* ^2^ = 22.65; *P*-value < 0.001).

**Table 2:** Social context of first alcohol experience (included only those with experience of alcohol consumption).

Social context	Boys				Girls			
	1995		2004		1995		2004	
	*N*	%	*N*	%	*N*	%	*N*	%
With parents	28	22	22	15	56	40	36	25
With friends	82	65	116	79	69	49	102	70
With somebody else (neighbours, relatives, etc.)	16	13	9	6	16	11	8	5

Total	126	100	147	100	141	100	146	100

*Chi-square*	*χ* ^2^ = 10 *P* = 0.031				*χ* ^2^ = 13.3 * P* = 0.001			

Gender differences in social context of first alcohol experience: 1995 (*χ* ^2^ = 9.54,  *P*-value = 0.008); 2004 (*χ* ^2^ = 4.66; *P*-value = *ns*).

**Table 3:** Frequency of alcohol consumption during the 12 months prior to survey.

Frequency of alcohol consumption	Boys				Girls			
	1995		2004		1995		2004	
	*N*	%	*N*	%	*N*	%	*N*	%
Weekly	23	13	48	28	11	6	26	15
Monthly	29	17	38	22	36	19	49	29
Few times a year	65	37	57	33	90	48	65	39
I did not consume	58	33	28	17	50	27	29	17

Total	175	100	171	100	187	100	169	100

*Chi-square*	*χ* ^2^ = 21 *P* < 0.001				*χ* ^2^ = 17 *P* = 0.001			

Gender differences in alcohol drinking during the past 12 months: 1995 (*χ* ^2^ = 9.22*, P-*value* = ns*); 2004 (*χ* ^2^ = 8.46*, P-*value* = 0.037).*

**Table 4:** Amount of participants who had consumed different types of alcohol beverages during the week prior to the survey (only those with experience of alcohol consumption are included).

Type of beverage	Boys			Girls		
	1995	2004	*P*-value	1995	2004	*P*-value
Beer	30%	32%	ns^∗^	8%	21%	0.006
Wine	8%	9%	ns^∗^	9%	7%	ns^∗^
Vodka	13%	13%	ns^∗^	6%	5%	ns^∗^
Long drink	NA^∗∗^	10%		NA^∗∗^	11%	

Total (*N*)	130	149		144	148	

^∗^Nonsignificant.

^∗∗^Not outlined in 1995.
